# Causal inference in real-world dementia research: a systematic review protocol

**DOI:** 10.1186/s13643-026-03179-w

**Published:** 2026-04-18

**Authors:** Yi Yang, Shivani Suresh, Jiamin Du, Rachel Brohier, Hongen Ma, Ivan Koychev, Vanessa Raymont

**Affiliations:** 1 Department of Psychiatry, University of Oxford, Oxford, UK; 2 NIHR Applied Research Collaboration Oxford and Thames Valley (NIHR ARC OxTV), Oxford Health NHS Foundation Trust, Oxford, UK; 3 Clinical Psychology Department, University of Edinburgh, Edinburgh, UK

**Keywords:** Dementia, Cognitive decline, Causal inference, Observational study, Systematic review

## Abstract

**Background:**

Dementia presents complex challenges for causal inference due to its multifactorial aetiology and slow, heterogeneous progression. Randomized controlled trials are often limited in their potential to fully address these challenges because of ethical and practical constraints. As the field evolves, observational studies incorporating advanced causal inference methods are increasingly used to estimate real-world effects in dementia research. However, the implementation of these methods varies widely and has not been systematically evaluated, with an emerging trend towards integration with techniques such as machine learning. This systematic review will critically examine how causal inference techniques are applied in dementia research, assess their methodological rigor, and identify trends, assumptions, and gaps that may inform future applications and methodological innovation in the field.

**Methods:**

Following PRISMA guidelines, searches will be conducted in MEDLINE, EMBASE, Web of Science, PsycINFO, Scopus, and the Cochrane Library for studies published between 1960 and 2024. Eligible studies will include observational designs that use causal inference methods to investigate outcomes such as cognitive decline, disease progression, and quality of life. Data extraction will capture study characteristics, methodological details, and key findings, with risk of bias assessed using ROBINS-I. A narrative synthesis will summarize qualitative results, and meta-analyses will be performed when methodological homogeneity permits.

**Discussion:**

This review will address a critical gap in the evaluation of the application of causal inference methods in real-world dementia research. By identifying methodological challenges, underlying assumptions, and emerging analytical techniques, it aims to strengthen research rigor and reproducibility and inform future methodological development, with potential implications for policy and practice in dementia care.

**Systematic review registration:**

PROSPERO (CRD42024619228).

**Supplementary Information:**

The online version contains supplementary material available at 10.1186/s13643-026-03179-w.

## Strengths and limitations of this study

- This review addresses a key gap by evaluating the current landscape and emerging trends in causal inference methodologies applied to dementia research using real-world data.
- By synthesizing evidence on causal inference methods in dementia research, this review aims to strengthen methodological rigor and inform the development of more robust, evidence-based research and practice.
- The review focuses on English-language, peer-reviewed observational studies, which may limit global representation and exclude insights from non-English publications, grey literature, and experimental research.

## Background

Dementia and related neurodegenerative diseases represent a growing global health concern, characterized by complex aetiologies, prolonged disease courses, and a significant societal burden [30, 41]. Dementia is classified under “Major Neurocognitive Disorder” in DSM-5 as a clinical syndrome marked by significant and persistent declines in cognitive functions—such as memory, reasoning, language, and executive functioning—that interfere with daily life and activities [1, 40]. Given that dementia arises from an interplay of genetic, environmental, and lifestyle factors, understanding which factors are causally linked to its onset and progression is essential for designing effective prevention and intervention strategies [37].

Randomized controlled trials (RCTs) are traditionally regarded as the gold standard for establishing causality, but their application in dementia research faces several limitations. The slow progression of the disease, combined with the difficulty of recruiting representative samples of older adults with comorbidities or frailty, often constrains the feasibility of RCTs [18, 29]. Additionally, randomizing participants to potentially harmful exposures or withholding beneficial treatments raises ethical concerns [12]. The prolonged follow-up periods required for dementia studies further increase the cost and complexity of conducting RCTs, making them impractical for many research questions [2, 25]. As a result, observational designs are becoming a critical alternative for investigating real-world exposures and outcomes in dementia research, where RCTs are unfeasible [5, 34].

However, traditional statistical methods used in observational studies, such as correlational or predictive analyses, are limited in their ability to establish causality. These approaches focus on identifying associations but often fail to address confounding, selection bias, and time-varying exposures, which are crucial for causal analysis [14, 33]. Furthermore, they cannot account for counterfactual scenarios, which limits their ability to rigorously estimate causal effects or test causal hypotheses [33]. While there is a growing trend in the use of machine learning techniques, which leverage advanced algorithms and computational power to identify patterns and generating hypotheses from large datasets, they too are limited in addressing biases and confounding without a causal framework [7]. Integrating the exploratory capabilities of machine learning with the rigor of causal inference techniques can provide robust and actionable insights into complex healthcare datasets, representing a promising direction for future research [7].

Causal inference methods are increasingly recognized as an essential framework for real-world observational studies, addressing the limitations of traditional statistical approaches and RCTs while driving advancements in research methodologies and evidence generation (Listl et al., 2016; [31]). These methods, such as propensity score matching, instrumental variables, and difference-in-differences, aim to mimic the rigor of RCTs by addressing confounding, selection bias, and unmeasured confounders in observational data [14, 21, 33]. For instance, propensity score methods (e.g., matching, weighting, stratification) address confounding in observational studies by summarizing multiple observed covariates into a single score, enabling the creation of comparable groups for estimating treatment effects. These methods offer a flexible and efficient way to reduce bias from measured confounders, but rely on the critical assumption of no unmeasured confounding [3, 4]. In contrast, instrumental variable analysis estimates causal effects in the presence of unmeasured confounding by using external variables—known as instruments—that influence the exposure but affect the outcome only through that exposure [15, 16, 20]. Meanwhile, difference-in-differences evaluates causal effects in observational studies by comparing changes in outcomes between treated and control groups over time. It allows for the estimation of treatment effects while accounting for unobserved, time-invariant confounding, under the assumption that both groups would have followed parallel trends in the absence of the intervention [35, 39]. Together, these methods provide essential tools for strengthening causal inference in observational dementia research, enabling more robust estimation of the effects of genetic, lifestyle, and environmental factors on dementia risk and progression.

Despite their advantages, causal inference methods require rigorous application and careful validation to ensure the reliability and validity of findings. A central challenge is addressing unmeasured confounding—methods like propensity scores can only balance observed covariates, and unmeasured variables cannot be corrected post hoc [13, 27, 43]. Proper model specification is also essential: statistical models must accurately reflect the relationships between exposures, confounders, and outcomes, as misspecification can lead to biased or invalid estimates [33, 38]. Instrumental variable methods, in particular, require strong instruments that are associated with the exposure, independent of unmeasured confounders, and influence the outcome solely through the exposure [11, 20]. Sensitivity analyses are critical for evaluating the robustness of causal estimates, and high-quality data that comprehensively captures relevant covariates is vital for drawing reliable conclusions [23, 36]. Finally, inconsistency in how these methods are applied and reported in dementia research raises concerns about comparability and generalizability across studies [6, 44].

Methodology-focused systematic reviews play a pivotal role in advancing research by critically evaluating the rigor, consistency, and appropriateness of applied analytical techniques. In dementia research, where unmeasured confounding, population heterogeneity, and the prolonged disease course pose persistent challenges, this review will assess how causal inference methods are being implemented to address these complexities. By identifying best practices, common methodological pitfalls, and underutilized or emerging techniques, it aims to support researchers in adopting more robust, transparent, and reproducible approaches. In doing so, the review contributes not only to strengthening the methodological foundations of future studies, but also to identifying emerging directions and innovations, thereby highlighting methodological developments worth watching in the evolving landscape of causal inference in dementia research. Ultimately, this systematic review aims to enhance the reliability and policy relevance of findings in dementia prevention and care.

## Systematic review

### Aim

This review aims to evaluate the application of causal inference methods in dementia research to support the generation of robust findings that can inform clinical and policy interventions. The review will address the following questions:

1. Topics: What causal factors have been evaluated for their relationship with dementia and neurodegenerative diseases in observational research?
2. Methods: Which causal inference methods (e.g., instrumental variables, difference-in-differences, target trial emulation, propensity score matching, g-methods) are applied in dementia research?
3. Datasets: How are causal inference methods tailored to different data types (e.g., longitudinal, cross-sectional, pooled datasets), and which approaches are suitable for specific study designs?
4. Causality and Assumptions: How do causal inference studies address challenges such as confounding, bias, and endogeneity, and how consistently are assumptions tested and reported?
5. Rigor and Heterogeneity: How do causal inference studies in dementia research address heterogeneity, such as population diversity and disease characteristics, and how do these variations influence the interpretation and the generalizability of findings?
6. Outcomes: What outcomes are examined using causal inference methods in dementia research, such as cognitive decline, disease progression, or intervention efficacy?
7. Emerging Methods and Gaps: What emerging technologies (e.g., machine learning) and methodologies are advancing causal inference in dementia research, and what gaps remain in their application?
8. Limitations: What practical challenges and systemic barriers influence the application of causal inference methods in observational dementia research, such as those related to implementation, scalability, and data quality?

## Methodology

Registration: This study protocol was registered with PROSPERO (CRD42024619228).

## Inclusion criteria

- Studies involving individuals with any form of dementia, such as Alzheimer’s disease, Parkinson’s disease, Lewy body dementia, cognitive decline (including those without an official diagnosis), or mild cognitive impairment (MCI), identified through validated screening tools or clinical diagnoses.
- Human studies only, with no demographic or geographic restrictions.
- Observational studies, including, case-control, cross-sectional, cohort, longitudinal, and pooled multi-cohort analyses, that investigate exposures or interventions related to neurodegenerative diseases or cognitive decline.
- Studies applying causal inference techniques, such as instrumental variables, difference-in-differences, propensity score matching, target trial emulation, G-methods, and regression discontinuity designs.
- Studies published in English language only.
- There is no restriction on exposures or interventions, such as studies examining the following:
- (1) Lifestyle factors: physical activity, diet, smoking, alcohol consumption, and sleep patterns.
- (2) Environmental factors: air pollution, occupational exposures, and access to healthcare.
- (3) Socioeconomic and psychological factors: education level, social isolation, and depression.
- (4) Biological and genetic factors: biomarkers (e.g., amyloid beta, tau proteins) and genetic predispositions (e.g., APOE ε4 allele).
-  (5) Medical and pharmacological interventions: use of medications (e.g., cholinesterase inhibitors), therapies (e.g., cognitive behavioural therapy), or surgical treatments (e.g., deep brain stimulation).
- (6) Digital and technological interventions: use of assistive technologies or telemedicine in managing neurodegenerative conditions.

## Exclusion criteria

- Studies focusing on conditions outside the scope of dementia, cognitive decline, mild cognitive impairment, or related neurodegenerative diseases.
- Studies conducted exclusively in experimental settings (e.g., RCTs) or involving purely experimental interventions that lack relevance to real-world observational contexts, unless they include an observational component where causal inference methods are applied.
- Animal or in vitro studies.
- Reviews, descriptive studies, or purely associational studies that do not employ causal inference methods.
- Theoretical models or simulations without empirical data.
- Studies lacking sufficient methodological detail to establish causal relationships between exposures/interventions and outcomes.

## Information sources

Peer-reviewed studies will be identified through comprehensive searches in electronic bibliographic databases, including MEDLINE/PubMed, EMBASE, Web of Science, PsycINFO, Scopus, and the Cochrane Library. These databases will be searched systematically to retrieve relevant studies from their inception to the present. Additionally, the reference lists of included studies will be reviewed to identify any further eligible studies that may not have been captured in the initial searches. The search strategy will be periodically updated to ensure the retrieval of the most recent and relevant literature in line with the systematic review’s objectives.

## Search strategy

The search strategy covers publications from 1960 to 2024 to capture a comprehensive timeline of relevant research. Only English language studies are included to ensure consistency in data extraction and analysis. An initial exploration of Medical Subject Headings (MeSH) and text words related to key concepts are conducted across all selected databases to identify terms relevant to the research questions. Titles, abstracts, and index terms of retrieved articles will be analysed iteratively to refine the strategy and enhance its sensitivity and specificity. Two independent reviewers will screen titles, abstracts, and full texts for eligibility, with disagreements resolved through discussion or consultation with a third reviewer. The search term framework is provided in Table 1. A detailed list of search terms and results is provided in the table in supplementary material and available on the Open Science Framework: https://osf.io/kyzvh. Table 1.

**Table 1:** Search term framework for identifying relevant studies in the literature search

Dementia terms	Causal inference terms	Exclusion terms
Dementia	Causal inference	Randomised control trial
Alzheimer*	Causal effect*	RCT
Cognitive decline	Causal estimation*	Review
Cognitive impairment	Counterfactual	
Corticobasal degeneration	Counterfactual framework	
Creutzfeldt Jakob syndrome	Inverse probability of treatment weighting	
Dementia with Lewy bodies	IPTW	
Lewy body dementia	Inverse probability weighting	
Frontotemporal lobar degeneration	IPW	
Frontotemporal dementia	Instrumental variable*	
Late onset dementia	Two-stage least squares	
Memory disorder*	2SLS	
Memory impair*	Generalized method of moments	
Neurodegenerat*	GMM	
Neurocognitive disorder*	Difference-in-differences	
neurodegenerative disease*	DiD	
Parkinson’s dementia	Fixed-effects model*	
Pick’s disease	Fixed-effects panel data analysis	
Mild cognitive impairment	Propensity score	
	Propensity score matching	
MCI	PSM Inverse probability weighted estimators G-method* G-computation G-formula Marginal structural model* MSM Marginal structural Cox model Mendelian Randomization Target trial emulation Regression discontinuity design* RDD Regression kink design* Structural causal model* SCM Directed acyclic graph* DAG Causal graphical models Causal mediation analysis Natural direct effect NDE Natural indirect effect NIE Synthetic control method* Comparative case study causal inference Causal machine learning Targeted maximum likelihood estimation TMLE Double machine learning DML Interrupted time series analysis	

## Outcomes

### There is no restriction on outcomes, and examples can be the following:

1. Cognitive Decline: Progressive deterioration in cognitive abilities, measured through standardized tests such as the Mini-Mental State Examination (MMSE [10];) and Montreal Cognitive Assessment (MoCA, [28]).
2. Disease Progression: Worsening symptoms or stages tracked through clinical scales like the Clinical Dementia Rating (CDR; [26]) or Hoehn and Yahr Scale [17], or biomarkers such as amyloid-beta and tau proteins.
3. Quality of Life: Well-being and functionality assessed using tools like the Quality of Life in Alzheimer’s Disease (QoL-AD; Logsdon et al., 24) and EuroQol 5 Dimensions (EQ-5D; EuroQol Group, [9]), including caregiver-reported assessments.
4. Mortality and Survival: Documented death due to neurodegenerative diseases or related complications, or time from diagnosis/symptom onset to death or study endpoint, analysed using survival models such as Kaplan–Meier curves.
5. Functional Decline: Loss of independence or ability to perform daily activities, assessed using scales such as Activities of Daily Living (ADL; [19]) and Instrumental Activities of Daily Living (IADL [22];).
6. Behavioural and Psychological Symptoms: Presence of conditions like depression, anxiety, and agitation, assessed with validated scales like the Neuropsychiatric Inventory (NPI [8];).
7. Neurological or Imaging Outcomes: Changes in brain structure or function measured through neuroimaging techniques like magnetic resonance imaging (MRI) and positron emission tomography (PET).
8. Social and Psychosocial Outcomes: Levels of social engagement or support, evaluated through self-reports, caregiver reports, or social network analyses.
9. Treatment Adherence and Adverse Effects: Treatments adherence and reporting of adverse effects, assessed through patient or caregiver reports and medical records.
10. Other relevant outcomes.

## Data management and study selection

Articles identified through the search process will be imported into systematic review software, Rayyan (https://www.rayyan.ai/), to ensure the process is rigorous. Duplicate entries will be identified and removed before proceeding with the review. A reference management software, such as Zotero, will be adopted to organize and manage citations. Two independent reviewers will screen the titles and abstracts of the retrieved articles to identify potentially eligible studies based on pre-defined inclusion and exclusion criteria. Full texts of these studies will then be assessed to confirm eligibility. Discrepancies at any stage will be resolved through discussion, and if consensus cannot be reached, a third reviewer will be consulted. The results of the selection process, along with the reasons for exclusion, will be documented and presented in a flow diagram following the Preferred Reporting Items for Systematic Reviews and Meta-Analyses (PRISMA) guidelines (Page et al., 32).

## Data extraction and recording

To ensure consistency and minimize errors, a standardized data extraction form will be developed using tools such as Excel or systematic review management software and piloted by the reviewers.

Data extraction will be conducted independently by two reviewers, with discrepancies resolved through discussion or consultation with a third reviewer when necessary. Data extraction will follow a detailed coding framework to ensure consistency, including study characteristics (e.g., author(s), publication year, country, study design and population details such as age, gender, diagnosis, and sample size), exposure/intervention descriptions (e.g., lifestyle factors, medical treatments), causal inference methods (e.g., instrumental variables, difference-in-differences, propensity score matching, target trial emulation), outcomes (e.g., cognitive decline, quality of life), key results (effect estimates, confidence intervals, sensitivity or robustness analyses, heterogeneity), study quality (e.g., confounding adjustments, handling of missing data, assumption tests, and risk of bias), emerging methods and gaps (e.g., machine learning, artificial intelligence), and practical challenges and barriers (e.g., computational complexity, scalability). The extracted findings will be presented using tools such as PRISMA flow diagrams, data extraction tables, and quality assessment forms, ensuring transparency and reproducibility of the review process.

## Quality appraisal

The risk of bias and quality of the included studies will be critically evaluated by two independent reviewers, with discrepancies resolved through discussion or, if necessary, consultation with a third reviewer. The ROBINS-I (Risk of Bias in Non-randomized Studies of Interventions) tool will be used to evaluate risk of bias. ROBINS-I assesses bias across seven key domains: confounding, selection of participants, classification of interventions, deviations from intended interventions, missing data, outcome measurement, and selective reporting, and evaluating how effectively studies address these issues. It categorizes risk of bias for each domain and overall as “low risk,” “moderate risk,” “serious risk,” or “critical risk,” based on predefined criteria. By comparing bias levels with those of well-conducted RCTs, ROBINS-I provides a structured framework for assessing the validity of causal estimates in observational research [42].

Additionally, a custom causal inference checklist will be employed to systematically evaluate key aspects of each causal inference method, such as the validity of assumptions (e.g., no unmeasured confounding, proper model specification) and the robustness of methods (e.g., sensitivity analyses, handling of bias), as outlined in Table 2. This checklist will complement the ROBINS-I tool by focusing specifically on the methodological rigor and application of causal inference techniques. The appraisal findings will be integrated into the discussion to provide a comprehensive evaluation of the strengths, limitations, and reliability of the included studies (Table 2).

**Table 2:** Diagnostics and sensitivity/robustness checks

Causal Inference Method	Key Assumptions/Considerations	Robustness Check
Instrumental variables	Instrument relevance, validity of exclusion restriction, and assessment about weak instruments	Overidentification tests, sensitivity analyses for exclusion restriction, and diagnostics for weak instruments
Difference-in-differences	Parallel trends assumption, adjustment for time-varying confounding, and handling of staggered adoption	Pre-treatment trend visualization, placebo tests, and robustness to alternative model specifications
Propensity score methods (e.g., matching)	Appropriate covariate inclusion, balance diagnostics post-matching, and assessment of overlap and common support	Standardized mean differences, sensitivity analyses for unmeasured confounding, and alternative propensity score models
G-methods (e.g., marginal structural models)	Correct model specification, stabilized weights, and adherence to the positivity assumption	Weight diagnostics, sensitivity to model misspecification, and sensitivity to potential unmeasured confounding
Target Trial Emulation	Specification of a clear trial design (eligibility, treatment, follow-up) and addressing immortal time bias	Alternative trial specifications, validation of key assumptions, and sensitivity to immortal time bias
Regression discontinuity design	Justification for the running variable, balance at the cutoff, and appropriate functional form	Placebo cutoffs, alternative bandwidths, and falsification tests for continuity
General causal estimation / counterfactual approaches	Clear definition of counterfactuals and handling of unmeasured confounding	Sensitivity analyses for confounding, alternative methods, and transparent reporting of limitations

## Quantitative synthesis (Meta-analysis)

Where sufficient data and methodological homogeneity are present, meta-analyses will be conducted to pool effect estimates for specific outcomes and causal inference methods. Statistical heterogeneity will be assessed using the *I*^2^ statistic and Cochran’s *Q* test. Subgroup analyses and meta-regression will explore sources of heterogeneity, such as study design, population characteristics, and data types. Meta-analyses will be conducted using software like R (e.g., *meta* or *metafor* packages) or Stata, and results will be visualized using forest plots.

## Discussion

This systematic review will provide a comprehensive synthesis of causal inference methods applied in observational studies of dementia and related neurodegenerative conditions. It will evaluate methods such as instrumental variables, difference-in-differences, G-methods, and propensity score-based approaches, with a focus on their ability to address confounding, bias, and other methodological challenges inherent to real-world data. The review will further examine how these methods are adapted to various data types, study designs, and population characteristics, highlighting their strengths, limitations, and appropriateness for different research contexts. By identifying common methodological challenges and critically assessing the assumptions underlying each approach, the review aims to support more rigorous, transparent, and reproducible causal inference in dementia research. Ultimately, its findings are expected to help bridge the gap between observational evidence and actionable insights, informing future research and methodological development, with potential implications for policy and practice in dementia care and prevention.

## Supplementary Information

Supplementary Material 1.

## Data Availability

Not applicable.

## Abbreviations

ADL: Activities of Daily Living (Katz et al., 1963)
APOE: Apolipoprotein E
CDR: Clinical Dementia Rating (Morris, 1993)
DSM-5: Diagnostic and Statistical Manual of Mental Disorders, Fifth Edition (American Psychiatric Association, 2013 [1])
EQ-5D: EuroQol 5 Dimensions (EuroQol Group, [9])
IADL: Instrumental Activities of Daily Living (Lawton & Brody, 1969)
MRI: Magnetic Resonance Imaging
MeSH: Medical Subject Headings
MCI: Mild Cognitive Impairment
MMSE: Mini-Mental State Examination (Folstein et al., 1975)
MoCA: Montreal Cognitive Assessment (Nasreddine et al., 2005)
NPI: Neuropsychiatric Inventory (Cummings et al., 1994)
PET: Positron Emission Tomography
PRISMA: ]Preferred Reporting Items for Systematic Reviews and Meta-Analyses (Page et al., [32])
QoL-AD: Quality of Life in Alzheimer’s Disease (Logsdon et al., 24)
RCT: Randomized Controlled Trial
ROBINS-I: Risk of Bias in Non-randomized Studies of Interventions (Sterne et al., 2016 [42])
