# Supplementary material for: Causal inference in real-world dementia research: a systematic review protocol
Source: Syst Rev. 2026 Apr 18;15:178. doi: 10.1186/s13643-026-03179-w (PMC13220476; doi:10.1186/s13643-026-03179-w)
Supplement: Supplementary file 1 — Supplementary Material 1. [file 13643_2026_3179_MOESM1_ESM.docx]

**Supplementary Material**

| **Number** | **Search Terms** | **Results** |
| --- | --- | --- |
| Cochrane Library | #1 MeSH descriptor: [Dementia] explode all trees  #2 MeSH descriptor: [Alzheimer Disease] explode all trees  #3 MeSH descriptor: [Cognitive Dysfunction] explode all trees  #4 MeSH descriptor: [Corticobasal Degeneration] explode all trees  #5 MeSH descriptor: [Creutzfeldt-Jakob Syndrome] explode all trees  #6 MeSH descriptor: [Lewy Body Disease] explode all trees  #7 MeSH descriptor: [Frontotemporal Dementia] explode all trees  #8 MeSH descriptor: [Memory Disorders] explode all trees  #9 MeSH descriptor: [Neurocognitive Disorders] explode all trees  #10 MeSH descriptor: [Pick Disease of the Brain] explode all trees  #11 MeSH descriptor: [Cognitive Dysfunction] explode all trees  #12 #1 OR #2 OR #3 OR #4 OR #5 OR #6 OR #7 OR #8 OR #9 OR #10 OR # 11  #13 (Causal inference):ti,ab,kw OR (Causal effect*):ti,ab,kw OR (Causal estimation*):ti,ab,kw OR ("counterfactual"):ti,ab,kw OR (Counterfactual framework):ti,ab,kw (Word variations have been searched)  #14 (Inverse probability of treatment weighting):ti,ab,kw OR (Inverse probability weighting):ti,ab,kw OR (Instrumental variable*):ti,ab,kw OR (Two-stage least squares):ti,ab,kw OR (Generalized method of moments):ti,ab,kw (Word variations have been searched)  #15 (Difference-in-differences):ti,ab,kw OR (Fixed-effects model*):ti,ab,kw OR (Fixed-effects panel data analysis):ti,ab,kw OR ("propensity score matching"):ti,ab,kw OR (Inverse probability weighted estimators):ti,ab,kw (Word variations have been searched)  #16 (G-method*):ti,ab,kw OR (G-computation):ti,ab,kw OR (G-formula):ti,ab,kw OR (Marginal structural model*):ti,ab,kw OR (Marginal structural Cox model):ti,ab,kw (Word variations have been searched)  #17 (Target trial emulation):ti,ab,kw OR (Regression discontinuity design*):ti,ab,kw OR (Regression kink design*):ti,ab,kw OR (Structural causal model*):ti,ab,kw OR (Directed acyclic graph*):ti,ab,kw (Word variations have been searched)  #18 (Causal graphical models):ti,ab,kw OR (Causal mediation analysis):ti,ab,kw OR (Natural direct effect):ti,ab,kw OR (Natural indirect effect):ti,ab,kw OR (Synthetic control method*):ti,ab,kw (Word variations have been searched)  #19 (Comparative case study causal inference):ti,ab,kw OR (Causal machine learning):ti,ab,kw OR (Targeted maximum likelihood estimation):ti,ab,kw OR (Double machine learning):ti,ab,kw OR (Interrupted time series analysis):ti,ab,kw (Word variations have been searched)  #20 ("randomised clinical trial"):ti,ab,kw OR (RCT):ti,ab,kw OR ("review"):ti,ab,kw (Word variations have been searched)  #21 #13 OR #14 OR #15 OR #16 OR #17 OR #18 OR #19  #22 #12 AND #21  #23 ("randomised clinical trial"):ti,ab,kw OR ("review"):ti,ab,kw (Word variations have been searched)  #24 #22 NOT #23 with Cochrane Library publication date Between Jan 1960 and Feb 2025 | 7,553 |
| PubMed | ("Dementia" OR "Alzheimer*" OR "Cognitive decline" OR "Cognitive impairment" OR "Corticobasal degeneration" OR "Creutzfeldt-Jakob syndrome" OR "Dementia with Lewy bodies" OR "Lewy body dementia" OR "Frontotemporal lobar degeneration" OR "Frontotemporal dementia" OR "Late onset dementia" OR "Memory disorder*" OR "Memory impair*" OR "Neurodegenerat*" OR "Neurocognitive disorder*" OR "Parkinson’s dementia" OR "Pick's disease" OR "Mild cognitive impairment" OR "MCI")  AND  ("Causal inference" OR "Causal effect*" OR "Causal estimation*" OR "Counterfactual" OR "Counterfactual framework" OR "Inverse probability of treatment weighting" OR "IPTW" OR "Inverse probability weighting" OR "IPW" OR "Instrumental variable*" OR "Two-stage least squares" OR "2SLS" OR "Generalized method of moments" OR "GMM" OR "Difference-in-differences" OR "DiD" OR "Fixed-effects model*" OR "Fixed-effects panel data analysis" OR "Propensity score" OR "Propensity score matching" OR "PSM" OR "Inverse probability weighted estimators" OR "G-method*" OR "G-computation" OR "G-formula" OR "Marginal structural model*" OR "MSM" OR "Marginal structural Cox model" OR "Target trial emulation" OR "Regression discontinuity design*" OR "RDD" OR "Regression kink design*" OR "Structural causal model*" OR "SCM" OR "Directed acyclic graph*" OR "DAG" OR "Causal graphical models" OR "Causal mediation analysis" OR "Natural direct effect" OR "NDE" OR "Natural indirect effect" OR "NIE" OR "Synthetic control method*" OR "Comparative case study causal inference" OR "Causal machine learning" OR "Targeted maximum likelihood estimation" OR "TMLE" OR "Double machine learning" OR "DML" OR "Interrupted time series analysis")  NOT  ("Randomized controlled trial" OR "RCT" OR "Review") | 2,971 |
| APA PsycInfo  Embase Classic+Embase  Ovid MEDLINE(R) | 1 Dementia.mp. [mp=ti, ab, hw, tc, id, ot, tm, mf, tn, dm, dv, kf, fx, dq, bt, nm, ox, px, rx, ui, sy, ux, mx] 528263  2 limit 1 to english language 484127  3 limit 2 to humans 452233  4 Alzheimer*.mp. [mp=ti, ab, hw, tc, id, ot, tm, mf, tn, dm, dv, kf, fx, dq, bt, nm, ox, px, rx, ui, sy, ux, mx] 609422  5 Cognitive decline.mp. [mp=ti, ab, hw, tc, id, ot, tm, mf, tn, dm, dv, kf, fx, dq, bt, nm, ox, px, rx, ui, sy, ux, mx] 108320  6 Cognitive impairment.mp. [mp=ti, ab, hw, tc, id, ot, tm, mf, tn, dm, dv, kf, fx, dq, bt, nm, ox, px, rx, ui, sy, ux, mx] 301532  7 Corticobasal degeneration.mp. [mp=ti, ab, hw, tc, id, ot, tm, mf, tn, dm, dv, kf, fx, dq, bt, nm, ox, px, rx, ui, sy, ux, mx] 7428  8 Creutzfeldt-Jakob syndrome.mp. [mp=ti, ab, hw, tc, id, ot, tm, mf, tn, dm, dv, kf, fx, dq, bt, nm, ox, px, rx, ui, sy, ux, mx] 8029  9 Dementia with Lewy bodies.mp. [mp=ti, ab, hw, tc, id, ot, tm, mf, tn, dm, dv, kf, fx, dq, bt, nm, ox, px, rx, ui, sy, ux, mx] 17411  10 Lewy body dementia.mp. [mp=ti, ab, hw, tc, id, ot, tm, mf, tn, dm, dv, kf, fx, dq, bt, nm, ox, px, rx, ui, sy, ux, mx] 4519  11 Frontotemporal lobar degeneration.mp. [mp=ti, ab, hw, tc, id, ot, tm, mf, tn, dm, dv, kf, fx, dq, bt, nm, ox, px, rx, ui, sy, ux, mx] 10336  12 Frontotemporal dementia.mp. [mp=ti, ab, hw, tc, id, ot, tm, mf, tn, dm, dv, kf, fx, dq, bt, nm, ox, px, rx, ui, sy, ux, mx] 38729  13 Late onset dementia.mp. [mp=ti, ab, hw, tc, id, ot, tm, mf, tn, dm, dv, kf, fx, dq, bt, nm, ox, px, rx, ui, sy, ux, mx] 818  14 Memory disorder*.mp. [mp=ti, ab, hw, tc, id, ot, tm, mf, tn, dm, dv, kf, fx, dq, bt, nm, ox, px, rx, ui, sy, ux, mx] 93708  15 Memory impair*.mp. [mp=ti, ab, hw, tc, id, ot, tm, mf, tn, dm, dv, kf, fx, dq, bt, nm, ox, px, rx, ui, sy, ux, mx] 52794  16 Neurodegenerat*.mp. [mp=ti, ab, hw, tc, id, ot, tm, mf, tn, dm, dv, kf, fx, dq, bt, nm, ox, px, rx, ui, sy, ux, mx] 422803  17 Neurocognitive disorder*.mp. [mp=ti, ab, hw, tc, id, ot, tm, mf, tn, dm, dv, kf, fx, dq, bt, nm, ox, px, rx, ui, sy, ux, mx] 27528  18 Parkinson's dementia.mp. [mp=ti, ab, hw, tc, id, ot, tm, mf, tn, dm, dv, kf, fx, dq, bt, nm, ox, px, rx, ui, sy, ux, mx] 292  19 Pick's disease.mp. [mp=ti, ab, hw, tc, id, ot, tm, mf, tn, dm, dv, kf, fx, dq, bt, nm, ox, px, rx, ui, sy, ux, mx] 3697  20 Mild cognitive impairment.mp. [mp=ti, ab, hw, tc, id, ot, tm, mf, tn, dm, dv, kf, fx, dq, bt, nm, ox, px, rx, ui, sy, ux, mx] 92639  21 MCI.mp. [mp=ti, ab, hw, tc, id, ot, tm, mf, tn, dm, dv, kf, fx, dq, bt, nm, ox, px, rx, ui, sy, ux, mx] 78986  22 1 or 4 or 5 or 6 or 7 or 8 or 9 or 10 or 11 or 12 or 13 or 14 or 15 or 16 or 17 or 18 or 19 or 20 or 21 1487199  23 Causal inference.mp. [mp=ti, ab, hw, tc, id, ot, tm, mf, tn, dm, dv, kf, fx, dq, bt, nm, ox, px, rx, ui, sy, ux, mx] 13837  24 Causal effect*.mp. [mp=ti, ab, hw, tc, id, ot, tm, mf, tn, dm, dv, kf, fx, dq, bt, nm, ox, px, rx, ui, sy, ux, mx] 23383  25 Causal estimation*.mp. [mp=ti, ab, hw, tc, id, ot, tm, mf, tn, dm, dv, kf, fx, dq, bt, nm, ox, px, rx, ui, sy, ux, mx] 212  26 Counterfactual.mp. [mp=ti, ab, hw, tc, id, ot, tm, mf, tn, dm, dv, kf, fx, dq, bt, nm, ox, px, rx, ui, sy, ux, mx] 8990  27 Counterfactual framework.mp. [mp=ti, ab, hw, tc, id, ot, tm, mf, tn, dm, dv, kf, fx, dq, bt, nm, ox, px, rx, ui, sy, ux, mx] 368  28 Inverse probability of treatment weighting.mp. [mp=ti, ab, hw, tc, id, ot, tm, mf, tn, dm, dv, kf, fx, dq, bt, nm, ox, px, rx, ui, sy, ux, mx] 9285  29 Inverse probability weighting.mp. [mp=ti, ab, hw, tc, id, ot, tm, mf, tn, dm, dv, kf, fx, dq, bt, nm, ox, px, rx, ui, sy, ux, mx] 6063  30 Instrumental variable*.mp. [mp=ti, ab, hw, tc, id, ot, tm, mf, tn, dm, dv, kf, fx, dq, bt, nm, ox, px, rx, ui, sy, ux, mx] 15133  31 Two-stage least squares.mp. [mp=ti, ab, hw, tc, id, ot, tm, mf, tn, dm, dv, kf, fx, dq, bt, nm, ox, px, rx, ui, sy, ux, mx] 1095  32 Generalized method of moments.mp. [mp=ti, ab, hw, tc, id, ot, tm, mf, tn, dm, dv, kf, fx, dq, bt, nm, ox, px, rx, ui, sy, ux, mx] 939  33 Difference-in-differences.mp. [mp=ti, ab, hw, tc, id, ot, tm, mf, tn, dm, dv, kf, fx, dq, bt, nm, ox, px, rx, ui, sy, ux, mx] 11039  34 Fixed-effects model*.mp. [mp=ti, ab, hw, tc, id, ot, tm, mf, tn, dm, dv, kf, fx, dq, bt, nm, ox, px, rx, ui, sy, ux, mx] 15776  35 Fixed-effects panel data analysis.mp. [mp=ti, ab, hw, tc, id, ot, tm, mf, tn, dm, dv, kf, fx, dq, bt, nm, ox, px, rx, ui, sy, ux, mx] 14  36 Propensity score.mp. [mp=ti, ab, hw, tc, id, ot, tm, mf, tn, dm, dv, kf, fx, dq, bt, nm, ox, px, rx, ui, sy, ux, mx] 138339  37 Propensity score matching.mp. [mp=ti, ab, hw, tc, id, ot, tm, mf, tn, dm, dv, kf, fx, dq, bt, nm, ox, px, rx, ui, sy, ux, mx] 67035  38 Inverse probability weighted estimators.mp. [mp=ti, ab, hw, tc, id, ot, tm, mf, tn, dm, dv, kf, fx, dq, bt, nm, ox, px, rx, ui, sy, ux, mx] 97  39 G-method*.mp. [mp=ti, ab, hw, tc, id, ot, tm, mf, tn, dm, dv, kf, fx, dq, bt, nm, ox, px, rx, ui, sy, ux, mx] 1216  40 G-computation.mp. [mp=ti, ab, hw, tc, id, ot, tm, mf, tn, dm, dv, kf, fx, dq, bt, nm, ox, px, rx, ui, sy, ux, mx] 1882  41 G-formula.mp. [mp=ti, ab, hw, tc, id, ot, tm, mf, tn, dm, dv, kf, fx, dq, bt, nm, ox, px, rx, ui, sy, ux, mx] 791  42 Marginal structural model*.mp. [mp=ti, ab, hw, tc, id, ot, tm, mf, tn, dm, dv, kf, fx, dq, bt, nm, ox, px, rx, ui, sy, ux, mx] 2940  43 Marginal structural Cox model.mp. [mp=ti, ab, hw, tc, id, ot, tm, mf, tn, dm, dv, kf, fx, dq, bt, nm, ox, px, rx, ui, sy, ux, mx] 136  44 Target trial emulation.mp. [mp=ti, ab, hw, tc, id, ot, tm, mf, tn, dm, dv, kf, fx, dq, bt, nm, ox, px, rx, ui, sy, ux, mx] 811  45 Regression discontinuity design*.mp. [mp=ti, ab, hw, tc, id, ot, tm, mf, tn, dm, dv, kf, fx, dq, bt, nm, ox, px, rx, ui, sy, ux, mx] 1395  46 Regression kink design*.mp. [mp=ti, ab, hw, tc, id, ot, tm, mf, tn, dm, dv, kf, fx, dq, bt, nm, ox, px, rx, ui, sy, ux, mx] 19  47 Structural causal model*.mp. [mp=ti, ab, hw, tc, id, ot, tm, mf, tn, dm, dv, kf, fx, dq, bt, nm, ox, px, rx, ui, sy, ux, mx] 156  48 Directed acyclic graph*.mp. [mp=ti, ab, hw, tc, id, ot, tm, mf, tn, dm, dv, kf, fx, dq, bt, nm, ox, px, rx, ui, sy, ux, mx] 4075  49 Causal graphical models.mp. [mp=ti, ab, hw, tc, id, ot, tm, mf, tn, dm, dv, kf, fx, dq, bt, nm, ox, px, rx, ui, sy, ux, mx] 41  50 Causal mediation analysis.mp. [mp=ti, ab, hw, tc, id, ot, tm, mf, tn, dm, dv, kf, fx, dq, bt, nm, ox, px, rx, ui, sy, ux, mx] 1945  51 Natural direct effect.mp. [mp=ti, ab, hw, tc, id, ot, tm, mf, tn, dm, dv, kf, fx, dq, bt, nm, ox, px, rx, ui, sy, ux, mx] 233  52 Natural indirect effect.mp. [mp=ti, ab, hw, tc, id, ot, tm, mf, tn, dm, dv, kf, fx, dq, bt, nm, ox, px, rx, ui, sy, ux, mx] 329  53 Synthetic control method*.mp. [mp=ti, ab, hw, tc, id, ot, tm, mf, tn, dm, dv, kf, fx, dq, bt, nm, ox, px, rx, ui, sy, ux, mx] 548  54 Comparative case study causal inference.mp. [mp=ti, ab, hw, tc, id, ot, tm, mf, tn, dm, dv, kf, fx, dq, bt, nm, ox, px, rx, ui, sy, ux, mx] 0  55 Causal machine learning.mp. [mp=ti, ab, hw, tc, id, ot, tm, mf, tn, dm, dv, kf, fx, dq, bt, nm, ox, px, rx, ui, sy, ux, mx] 108  56 Targeted maximum likelihood estimation.mp. [mp=ti, ab, hw, tc, id, ot, tm, mf, tn, dm, dv, kf, fx, dq, bt, nm, ox, px, rx, ui, sy, ux, mx] 573  57 Double machine learning.mp. [mp=ti, ab, hw, tc, id, ot, tm, mf, tn, dm, dv, kf, fx, dq, bt, nm, ox, px, rx, ui, sy, ux, mx] 63  58 Interrupted time series analysis.mp. [mp=ti, ab, hw, tc, id, ot, tm, mf, tn, dm, dv, kf, fx, dq, bt, nm, ox, px, rx, ui, sy, ux, mx] 8477  59 23 or 24 or 25 or 26 or 27 or 28 or 29 or 30 or 31 or 32 or 33 or 34 or 35 or 36 or 37 or 38 or 39 or 40 or 41 or 42 or 43 or 44 or 45 or 46 or 47 or 48 or 49 or 50 or 51 or 52 or 53 or 54 or 55 or 56 or 57 or 58 244136  60 1 and 2 and 3 and 59 | 3291 |
